# The burden of unrecognised chronic kidney disease in patients with type 2 diabetes at a county hospital clinic in Kenya: implications to care and need for screening

**DOI:** 10.1186/s12882-020-1705-3

**Published:** 2020-02-28

**Authors:** Frederick C. F. Otieno, Elijah N. Ogola, M. W. Kimando, Kenn Mutai

**Affiliations:** 1 Department of Clinical Medicine and Therapeutics, College of Health Sciences, University of Nairobi, Box 19676-00202, Nairobi, Kenya; 2 Division of Medicine, Kenyatta National Hospital, Box 20723-00202, Nairobi, Kenya; 3 Nyeri County Hospital, Nyeri, Kenya

## Abstract

**Background:**

Chronic Kidney Disease (CKD) in patients with type 2 diabetes enhances the cardiovascular risk profiles and disease, and is a strong predictor of progression to end-stage kidney disease. Early diagnosis is encouraged for referral to specialist kidney care to initiate active management that would optimize outcomes including forestalling progression to end-stage kidney disease.

This study was conducted in a regional referral public health facility in Central Kenya with a high prevalence of type 2 diabetes. It was aimed at finding out the burden of undiagnosed chronic kidney disease in their clinic of ambulatory patients with type 2 diabetes who dwell mainly in the rural area.

**Methods:**

A cross-sectional study was conducted at the out-patient of Nyeri County hospital. A total of 385 patients were enrolled over 5 months. Informed consent was obtained and clinical evaluation was done, a spot sample of urine obtained for albuminuria and venous blood drawn for HbA1c, Lipids and serum creatinine. Estimated GFR (eGFR) was calculated using the Cockroft-Gault equation. Chronic kidney disease (CKD) was classified on KDIGO scale. Albuminuria was reported as either positive or negative. Descriptive statistics for data summary and regression analysis were employed on SPSS v23.

**Results:**

A total of 385 participants were included in the study, 252 (65.5%) were females. There were 39.0% (95%CI 34.3–44.2) patients in CKD/KDIGO stages 3, 4 and 5 and 32.7% (95%CI, 27.8–37.4) had Albuminuria. The risk factors that were significantly associated with chronic kidney disease/KDIGO stages 3, 4 and 5 were: age > 50 years, long duration with diabetes > 5 years and hypertension. Employment and paradoxically, obesity reduced the odds of having CKD, probably as markers of better socio-economic status.

**Conclusion:**

Unrecognized CKD of KDIGO stages 3,4 and 5 occurred in over 30 % of the study patients. The risk factors of hypertension, age above 50, long duration of diabetes should help identify those at high risk of developing CKD, for screening and linkage to care. They are at high risk of progression to end-stage kidney disease and cardiovascular events. The imperative of screening for chronic kidney disease is availing care in publicly-funded hospitals.

## Background

Approximately 40% of patients with diabetes, in their lifetime, develop Diabetes Kidney Disease defined by impaired or falling Glomerular Filtration Rate and/or albuminuria [1]**.** Additional development of CKD on diabetes greatly enhances the risk of cardiovascular events and poor cardiovascular outcomes [2–5].

.The UKPDS [6] demonstrated that after median follow up of 15 years, 38% of the study patients previously with normo-albuminuria and 29% normal GFR developed albuminuria and impaired GFR respectively. The time span to kidney events may certainly be shorter in real-life and non-trial clinical care conditions, probably more so in sub-Saharan Africa where the challenges to organization and provision of care limit achievement of optimal care for so many patients.

CKD in type 2 diabetes is a subtle disease in early phases, but when it becomes manifest, it is severe. Patients seek care when prompted by symptoms. However, access to care is considered insufficient until those at high risk of developing CKD are screened and case identification established as a standard of care.

That CKD is defined by albuminuria and/or impaired eGFR is important because they are relatively easy to measure though not routinely done or reported. In Kenya, indeed in sub-Saharan Africa, early and timely case-finding and access to care are challenges occasioned by scarce resources and non-integrated healthcare systems which can still be overcome. End-stage kidney disease is much worse: it is costly [7, 8] and carries high mortality [9], therefore secondary prevention strategies should be strengthened.

The context of the study is that public hospitals have challenges of healthcare provision at the level of clinical staff and the care support within the facilities, especially laboratories and pharmacies. Therefore, it is presumable that secondary prevention is sub-optimal in the patients who use such facilities.

## Methods

### Study design

A cross-sectional analysis of adult patients with type 2 diabetes without overt complications or co-morbidities attending the out-patient diabetic clinic.

### Sample size determination

A minimum sample size of 295 was determined using Fischer’s formula, *N* = 1/d^2^ * (Z^2^ x P (1-P)), *N* = Sample size, Z = 1.96 (95% confidence interval), P = Estimated proportion of chronic kidney disease in type 2 diabetes patients = 26% [from a previous study [10],], d = Margin of error (precision error) = ±5%, substituting into the formula, *N* = 295. However, we enrolled 385 participants to address the objective of adequacy of control of cardiovascular risk factors in the same target population, already published [11].

### .Study setting

The study site is a public hospital in Central Kenya, a region of higher prevalence of type 2 diabetes. The diabetes clinic is run every Friday morning each week except on public holidays. About 100 patients with either type 1 or 2 diabetes mellitus are seen on each clinic day by a Medical officer, registered Nursing staff and occasionally, a consultant physician. Booked patients receive clinical reviews that include but not limited to, weight and Blood Pressure measurements, diabetes education and blood glucose assays. Serum lipids, creatinine, and Albuminuria tests are not routine for patients on a clinic day.

### Study population

Adults with file diagnosis of type 2 diabetes, aged > 30 years, on follow-up in the clinic for not less than 6 months and on anti-diabetic treatment.

### Exclusion criteria

We excluded patients who were/had type 1 diabetes mellitus or type 2 diabetes that had been hospitalized in the previous 1 month prior to the index clinic visit, file diagnosis of heart failure or chronic kidney disease, secondary diabetes mellitus. Those patients with type 2 diabetes who declined to give consent to participate were also excluded.

They were enrolled on consecutive clinic days over nearly five (5) months between November, 2014 and early March 2015.

### Sampling, recruitment and data collection

**Fig. 1:**
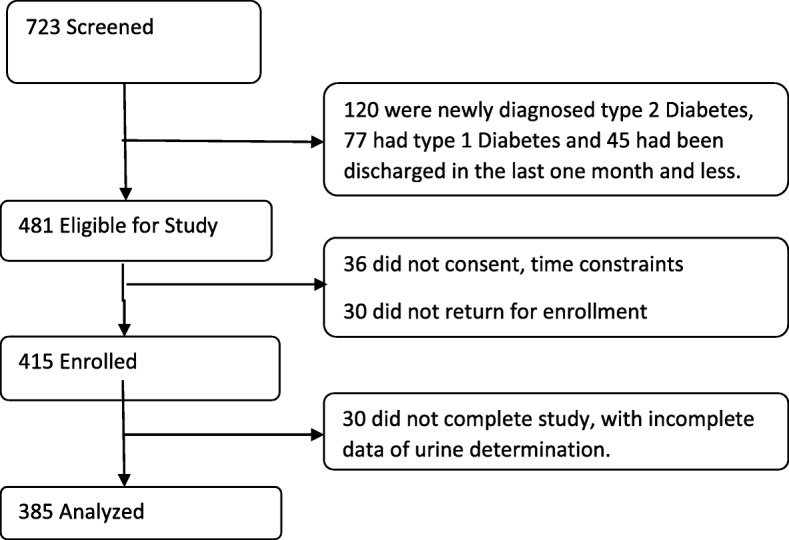
A flow chart of recruitment and enrolment of subjects into the study

The investigators went through the files before the start of the day’s clinic session and selected all eligible patients. The eligible participants, on each morning of the clinic day, were assigned random numbers and then systematically selected. Each patient who met the inclusion criteria was given full explanation of the study and enrolled after giving written informed consent. Summary of the recruitment flow is shown on Fig. 1.

Figure 1 summarizes the recruitment flow of the study subjects. Three hundred and eighty-five (385) patients with type 2 diabetes in ambulatory setting were included in the study.

We documented socio-demographic information of age, gender, occupation, level of education, duration of diabetes, and treatment history (of hyperglycaemia, hypertension, and lipid disorder) and any other illnesses. Chart/file review was done to corroborate the information. HIV status was enquired and all the patients interviewed reported a negative status. Marital status, level of formal education, alcohol intake and cigarette smoking were recorded.

Full clinical examination was done; weight and height were measured and BMI calculated as Weight (kg) divided by square of Height (m^2^). Waist and hip circumferences were also measured in centimeters. Blood Pressure was determined using a manual mercury sphygmomanometer, the standard way, after the patient had rested for not less than ten (10) minutes. Diagnosis of hypertension was made at BP ≥140/90 mmHg, and classified per JNC 8 [12].

### Laboratory

A 10-ml venous blood sample was drawn from the antecubital fossa, from which a 2-ml blood sample was processed in an EDTA-anti-coagulated bottle for HbA_**1**_c determination. **HBA1c** was processed by glycol-hemoglobin ion exchange resin method from *ERBA MANNHEIM Gmbh* at the laboratory. Any value of HbA1c > 7.0% was considered sub-optimal control. The other 8-ml blood sample was processed in a plane bottle and transported for automated assay of serum creatinine and lipids.

Lipid profile was analyzed using *Human Gmbh* kit. Total cholesterol was measured using the CHOD-PAP method based on Trinders Methodology, a calorimetric, enzymatic test for cholesterol with lipid clearing factor. HDL cholesterol was measured using human cholesterol liqui-color Phosphatungstic Acid method, end-point kit. *GPO-PAPA METHOD*, a colorimetric, enzymatic method with glycerophosphate oxidase was employed to assay the triglycerides. LDL-cholesterol was computed from the formula: [LDL-chol] = [Total chol] – [HDL-chol] - ([TG]/2.2) where all concentrations are given in mmol/L. LDL-Cholesterol above 2.0 mmol/L was considered higher risk level.

From the serum creatinine results, estimated glomerular filtration rate was calculated on Cockroft-Gault formula:

$$ \mathrm{GFR}-\mathrm{Cockcroft}-\mathrm{Gault}=\frac{\left(140-\mathrm{age}\times \mathrm{body}\kern0.17em \mathrm{weight}\right)}{\mathrm{serum}\kern0.17em \mathrm{creatinine}\times 0.814\;\mathrm{for}\kern0.17em \mathrm{males}} $$

The results were the multiplied by 0.85 for females; where age is in years, weight in kg and serum creatinine in micromol/L. [13].

SPOT urine was obtained from each participant in the morning of the clinic day for Albuminuria determination semi-quantitatively as **Urinary albumin to creatinine ratio.***CLINITEK Microalbuminuria reagent strips* were used, where CLINITEK Micral-2 Strips were dipped in freshly-voided urine sample to provide semi-quantitative albumin-to-creatinine ratio results in 1 minute.

A single spot urine test has been demonstrated as reliable [14], and has been shown to compare well with the gold standard, 24-h urinary albuminuria [15].

### Statistical analysis

We then summarized the data and categorical data (gender, age categories, socio-demographics, habits of alcohol-intake, cigarette-smoking and other clinico-laboratory categories-especially eGFR categories/stages) were summarized as proportions. The continuous data were presented as mean (+/−SD), median and interquartile range. Differences in mean +/−SD between the socio-demographic and clinical characteristics of the patient groups were analyzed using student t-tests. Chi-square and odds ratios (OR), where applicable, were used for association of variables with chronic kidney disease. Logistic regressions were done on bivariate analysis where/when *p*-value was significant, *p* < 0.05. The odds ratios (OR) were expressed in 95% confidence intervals and p-value was taken as significant at *p* ≤ 0.05. These were analyzed on the statistical software, SPSS version 20.0.

## Results

A total of 385 participants were included in the study, 252 (65.5%) were females.

The characteristics of the study population are summarized in the Table 1 below.

**Table 1:** Clinical and laboratory characteristics by gender of the study patients

Variable	Overall(*N* = 385) N (%)	Female(*N* = 252) N (%)	Male (*N* = 133) N (%)	OR (95% CI)	*P* value
Age, years, mean(SD)	63.3	62.1 (12.0)	65.7 (13.7)	–	**0.006**
BMI,kg/m^2^,mean (SD) Categories, n (%)	26.7 (4.6)				
Underweight- (< 18.5 kg/m^2^)	6 (1.6)	4 (1.6)	2 (1.5)	1.5 (0.3–8.3)	0.660
Normal- (18.5-25 kg/m^2^)	139 (36.1)	80 (31.7)	59 (44.4)	1.0	–
Overweight- (25–29.9 kg/m^2^)	154 (40.0)	102 (40.5)	52 (39.1)	1.4 (0.9–2.3)	0.127
Obese- (≥30 kg/m^2^)	86 (22.3)	66 (26.2)	20 (15.0)	2.4 (1.3–4.4)	**0.004**
Waist Circumference, cm, mean (SD) Categories, n (%)	92.5 (22.0)				
Undesirable, > 102 cm(M)/> 88 cm(F)	224 (58.2)	171 (67.9)	53 (39.8)	3.2 (2.1–4.9)	**< 0.001**
Normal	161 (41.8)	81 (32.1)	80 (60.2)	1.0	
Hypertension, BP > 140/90 mmHg					
Hypertensive, n (%)	191 (49.6)	196 (77.8)	99 (74.4)	1.2 (0.7–2.0)	0.461
Normal BP	194 (50.4)	56 (22.2)	34 (25.6)	1.0	
Glycemic control, HbA1c (%)					
Glycaemia, HbA1c %, mean(SD)	8.1 (2.8)	8.3 (3.0)	7.9 (2.7)	–	0.181
Poor control, HbA1c > 7.0	233 (60.5)	154 (61.1)	79 (59.4)	1.1 (0.7–1.6)	0.744
Optimal control, HbA1c ≤ 7.0	152 (39.5)	98 (38.9)	54 (40.6)	1.0	
Total cholesterol, mean (SD), mmol/L Categories, n (%)	4.6 (1.2)	4.9 (1.2)	4.2 (1.1)	–	**< 0.001**
High> 4.120	88 (22.9)	69 (27.4)	19 (14.3)	2.3 (1.3–4.0)	**0.004**
Optimal≤4.120	297 (77.1)	183 (72.6)	114 (85.7)	1.0	
HDL-cholesterol, mean (SD),mmol/L Categories, n (%)	1.3 (0.9)	1.4 (0.3)	1.3 (1.5)	–	0.719
Low≤1.0	80 (20.8)	39 (15.5)	41 (30.8)	0.4 (0.2–0.7)	**< 0.001**
Optimal> 1.0	305 (79.2)	213 (84.5)	92 (69.2)	1.0	
Triglycerides, mean (SD), mmol/L Categories, n (%)	1.7 (1.0)	1.7 (1.0)	1.7 (1.1)	–	0.873
High > 1.7	210 (54.5)	135 (53.6)	75 (56.4)	0.9 (0.6–1.4)	0.597
Optimal≤1.7	175 (45.5)	117 (46.4)	58 (43.6)	1.0	
LDL-cholesterol, mean(SD, mmol/L Categories, n (%)	2.4 (0.9)	2.6 (0.9)	2.2 (0.9)	–	**< 0.001**
High> 2.0	297 (77.1)	206 (81.7)	91 (68.4)	2.1 (1.3–3.4)	**0.003**
Optimal≤2.0	88 (22.9)	46 (18.3)	42 (31.6)	1.0	
CKD/(KDIGO categories)					
G1, eGFR > 90 ml/min/m^2^	77 (20.0)	53 (21.0)	24 (18.0)	1.0	
G2, eGFR 60–90	158 (41.0)	106 (42.1)	52 (39.1)	0.9 (0.5–1.7)	0.789
G3a eGFR 59–45	81 (21.0)	48 (19.0)	33 (24.8)	0.7 (0.3–1.3)	0.211
G3b eGFR 44–30	48 (12.5)	30 (11.9)	18 (13.5)	0.8 (0.4–1.6)	0.467
G4 eGFR 29–15	18 (4.7)	13 (5.2)	5 (3.8)	1.2 (0.4–3.7)	0.779
G5 eGFR < 15	3 (0.8)	2 (0.8)	1 (0.8)	0.9 (0.1–10.5)	0.937
Albuminuria status					
Albuminuria present	126 (32.7)	80 (31.7)	46 (34.6)	0.9 (0.6–1.4)	0.572
NO Albuminuria	259 (67.3)	172 (68.3)	87 (65.4)	1.0	

Table 1 shows socio-demographic and treatment information of the study subjects. The population mean age was 63.3 years, IQR of 56-71 years. The males were significantly older, 65.7(13.7) years than the females, 62.1(12.0) years. The proportion of females with obesity (BMI ≥ 30 kg/m^2^) was 26.2%, compared to males at 15.0%, and similarly, more females than males had obesity when waist circumference was used. The bold *p*-values depict statistically significant differences between males and females.

Two hundred and thirty-three, 60.5% of the study subjects had poor glycaemic control and 191 (49.6%) had hypertension, of whom more than 75.0% of them were poorly controlled. The proportion of males and females with optimal control of hypertension and glycaemia were similar. However, the lipid parameters of total cholesterol and LDL-cholesterol were higher and HDL-cholesterol lower in the females.

The proportions of the study subjects with CKD in the various KDIGO stages (by eGFR) were: **G1** (> 90 ml/min/1.73m^2^)- 20%, **G2** (60-90ml/min/1.73m^2^) - 41%, **G3a** (45-60 ml/min/1.73m^2^) - 21% and **G3b** (30-45 ml/min/1.73m^2^) -12.5%, **G4** (15-30ml/min/1.73m^2^) - 4.7%, and **G5** (< 15 ml/min/1.73m^2^) - 0.8%. One hundred and fifty, 39.0% of the study patients had eGFR< 60 ml/min/1.73m^2^ while one hundred and twenty-six, 32.7% of them had Albuminuria. There were no gender differences in the proportions of subjects with albuminuria and those in the various CKD/KDIGO stages.

Table 2 shows treatment choices of the study patients: Over two-thirds, 68.1%, were on oral agents-only and 29.1% were on insulin-based therapy, either as combination with oral agents (12.0%) or insulin-only (17.1%). Use of ACEi/ARBs for hypertension treatment was documented in 69.0%. Over 80% of the subjects made four or more clinic visits in the previous 12 months.

**Table 2:** Treatment Information of the study patients

Variable	Proportion, N (%)
Diabetes mellitus treatment	
Diet-only	11 (2.9)
Oral Glucose-lowering Agents(OGLAs)-only	262 (68.1)
Insulin-only	66 (17.1)
Insulin combined with Oral Glucose-lowering Agents	46 (12.0)
Other co-medications used regularly by the subjects	
Anti-platelets	45 (11.7)
Statins	48 (12.5)
Anti-platelets and statins	108 (28.1)
Anti-hypertensive drugs	295 (76.6)
- ACEi/ARBs	204 (69.0)
Frequency of clinic attendance in the last 12 months	
2–3	68 (17.7)
4–5	292 (75.9)
6 and above	25 (6.5)

*ACEi* Angiotensin Converting Enzyme inhibitors, *ARBs* Angiotensin Receptor Blockers

Table 3 shows bivariate analysis of subjects in the early Chronic Kidney Disease (CKD) stages 1 and 2 compared to those with advanced CKD stages 3, 4 and 5. The mean age of the patients with advanced Chronic Kidney Disease (eGFR< 60 **ml/min/1.73m**^**2**^) was 70.8 years compared with 58.6 years of those in early CKD stages 1 and 2. The subjects in early CKD stages 1 and 2 had markers of better socio-economic status (namely, higher level of formal education and employment) than their counterparts with advanced CKD stages 3, 4 and 5. The mean SBP was higher (148.8 mmHg vs. 140.3 mmHg), and duration of diabetes was longer (11.0 versus 5.0 years) in patients with advanced CKD/KDIGO stages 3, 4 and 5 compared to those in early CKD/KDIGO stages 1 and 2. Gender did not increase the odds of occurrence of advanced CKD stages 3, 4 and 5, as the proportions were not significantly different between females (36.9%) and the males (42.9%), (*p* > 0.05).

**Table 3:** Bivariate analysis of factors associated with chronic kidney disease in the study subjects

Variable	Chronic Kidney Disease, CKD /KDIGO classification		OR (95% CI)	*P* value
	Stage 3–5	Stage 1–2		
Age, mean (SD), years	70.8 (8.8)	58.6 (11.5)	–	**< 0.001**
Age category, years				
≤ 50	0 (0.0%)	54 (100.0%)	–	**< 0.001**
> 50	150 (45.3%)	181 (54.7%)		
Gender				
Female	93 (36.9)	159 (63.1)	0.8 (0.5–1.2)	0.255
Male	57 (42.9)	76 (57.1)	1.0	
Marital status				
Single, unmarried	2 (9.1)	20 (90.9)	1.0	
Married.	96 (37.2)	162 (62.8)	5.9 (1.4–25.9)	**0.018**
Widowed	52 (51.0)	50 (49.0)	10.4 (2.3–46.8)	**0.002**
Separated	0 (0.0)	3 (100.0)	–	0.999
Level of formal education				
None	29 (56.9)	22 (43.1)	1.0	
Primary school(1-7 yrs)	87 (38.3)	140 (61.7)	0.5 (0.3–0.9)	**0.017**
Secondary school(8-12 yrs)	26 (28.9)	64 (71.1)	0.3 (0.2–0.6)	**0.001**
Tertiary, > 12 yrs. in school	8 (47.1)	9 (52.9)	0.7 (0.2–2.0)	0.483
Employment status				
Unemployed	56 (44.8)	69 (55.2)	1.0	
Employed	3 (13.0)	20 (87.0)	0.2 (0.1–0.7)	**0.009**
Self-employed	47 (31.5)	102 (68.5)	0.6 (0.3–0.9)	**0.025**
Retired	44 (50.0)	44 (50.0)	1.2 (0.7–2.1)	0.454
Cigarette smoking				
Smoker	38 (41.3%)	54 (58.7%)	1.1 (0.7–1.8)	0.597
Non-smoker	112 (38.2%)	181 (61.8%)	1.0	
Duration of diabetes, years, median (IQR)	11.0 (5.0–18.0)	5.0 (2.0–11.0)	–	**< 0.001**
Duration of disease, categories, years				
> 5	105 (48.2%)	113 (51.8%)	2.6 (1.7–4.1)	**< 0.001**
≤ 5	41 (26.1%)	116 (73.9%)	1.0	
LDL-cholesterol, mmol/L				
High> 2.0	116 (39.1)	181 (60.9)	1.0 (0.6–1.7)	0.943
Normal ≤2.0	34 (38.6)	54 (61.4)	1.0	
Obesity				
Obese, BMI ≥30 kg/m^2^	17 (19.8)	69 (80.2)	0.3 (0.2–0.5)	**< 0.001**
Not obese, BMI < 30 kg/m^2^	133 (44.5)	166 (55.5)	1.0	
Hypertension, BP > 140/90 mmHg				
Hypertensive	91 (47.6)	100 (52.4)	2.1 (1.4–3.2)	**0.001**
Normal BP	59 (30.4)	135 (69.6)	1.0	
Systolic BP,mean (SD) mmHg	148.8 (25.6)	140.3 (20.6)	–	**< 0.001**
Diastolic BP,mean (SD) mmHg	81.9 (12.1)	81.2 (10.9)	–	0.579
Glycemic control				
Poor (HbA1c > 7.0%)	86 (36.9)	147 (63.1)	0.8 (0.5–1.2)	0.307
Good (HbA1c ≤ 7.0%)	64 (42.1)	88 (57.9)	1.0	

The patients in either of the CKD groups had similar glycaemic control and mean diastolic blood pressure. Obesity (BMI ≥ 30 kg/m^2^) paradoxically reduced the odds of having advanced CKD (eGFR< 60 ml/min/1.73m^2^).

Table 4 shows logistic regression of the risk factors of higher stages of CKD (eGFR< 60 ml/min/1.73m^2^) and their adjusted Odds Ratios (OR) that were significant at bivariate analysis. Age above 50 years, longer duration of diabetes > 5 years, systolic blood pressure ≥ 140 mmHg and obesity (BMI ≥ 30 kg/m^2^) were significant determinants of Chronic Kidney Disease (eGFR< 60 ml/min/1.73m^2^).

**Table 4:** Logistic regression model of the predictors of Chronic Kidney Disease (CKD) in the study subjects

Variable	Adjusted odds ratios			*p*- value
	Odds ratio	95% C.I.		
		Lower	Upper	
Age, above 50 years	1.12	1.09	1.16	**< 0.001**
Duration of diabetes (> 5 years)	1.98	1.17	3.36	**0.012**
Duration of diabetes, years	1.05	1.05	1.08	**0.005**
Obesity, BMI ≥30 kg/m^2^	0.24	0.12	0.47	**< 0.001**
Systolic blood pressure, SBP ≥ 140 mmHg	1.014	1.003	1.025	**0.012**
Hypertension present	2.3	1.2	4.5	**0.015**

Age, marital status, level of formal education, employment status, duration of diabetes in years, obesity of BMI ≥30 kg/m^2^ and hypertension were included in the logistic regression model. Age, duration of diabetes above 5 years, hypertension (as both continuous and categorical variables) were found to increase the odds of having CKD/KDIGO stages 3 to 5, but obesity (by BMI) mitigated the risk of having Chronic Kidney Disease in the study patients.

Table 5 shows risk factor loading and the change in the odds of having advanced stages of Chronic Kidney Disease (eGFR< 60 ml/min/1.73m^2^). Hypertension alone increased the odds by three times (×3), but addition of glycaemic control (HbA1c), age above 50 years, duration of diabetes > 5 years and cigarette-smoking increased the odds by six times (× 6).

**Table 5:** Logistic regression of risk factors and loading on patients with chronic kidney disease in the study

Variable	Chronic Kidney Disease (CKD)	Normal, non-CKD	OR (95% CI)	*P* value
Risk factor loading,				
0-Normal Blood Pressure	20 (22.2%)	70 (77.8%)	1.0	
1-Hypertension (HTN)	53 (47.3%)	59 (52.7%)	3.1 (1.7–5.8)	< 0.001*
2-HTN + HbA1c > 7.0%	20 (42.6%)	27 (57.4%)	2.6 (1.2–5.6)	0.014*
3-HTN + HbA1c + LDL > 2.0 mmol/L	0 (0.0%)	13 (100.0%)	–	0.999
4-HTN + A1c + LDL + Age + Dur < 5 yr	17 (34.7%)	32 (65.3%)	1.9 (0.9–4.0)	0.114
5-HTN + HbA1c + LDL + Age + Dur ≥ 5 yr	29 (50.9%)	28 (49.1%)	3.6 (1.8–7.4)	< 0.001*
6-HTN + A1c + LDL + Age + Dur > 5 yr + Cig	11 (64.7%)	6 (35.3%)	6.4 (2.1–19.5)	0.001*
7- ACEI/ARB-use				
Yes	111 (41.6)	156 (58.4)	1.4 (0.9–2.3)	0.114
No	39 (33.1)	79 (66.9)	1.0	

*CKD* Chronic kidney disease, stages 3 to 5, Age = Age > 50 years, *Dur* Duration of diabetes, *Cig* Cigarette-smoking, *HTN* Hypertension, *LDL* Low Density Lipoprotein Cholesterol. The addition of LDL, Age and Duration of diabetes equal to/above 5 yrs did not enhance risk of CKD significantly in the risk factor loading model

Abbreviations. **CKD** - Chronic Kidney Disease, **KDIGO** - Kidney Disease. Improving Global Outcomes. **ACEi/ARBs**- Angiotensin Converting Enzyme inhibitors/Angiotensin Receptor Blockers.

**Fig. 2:**
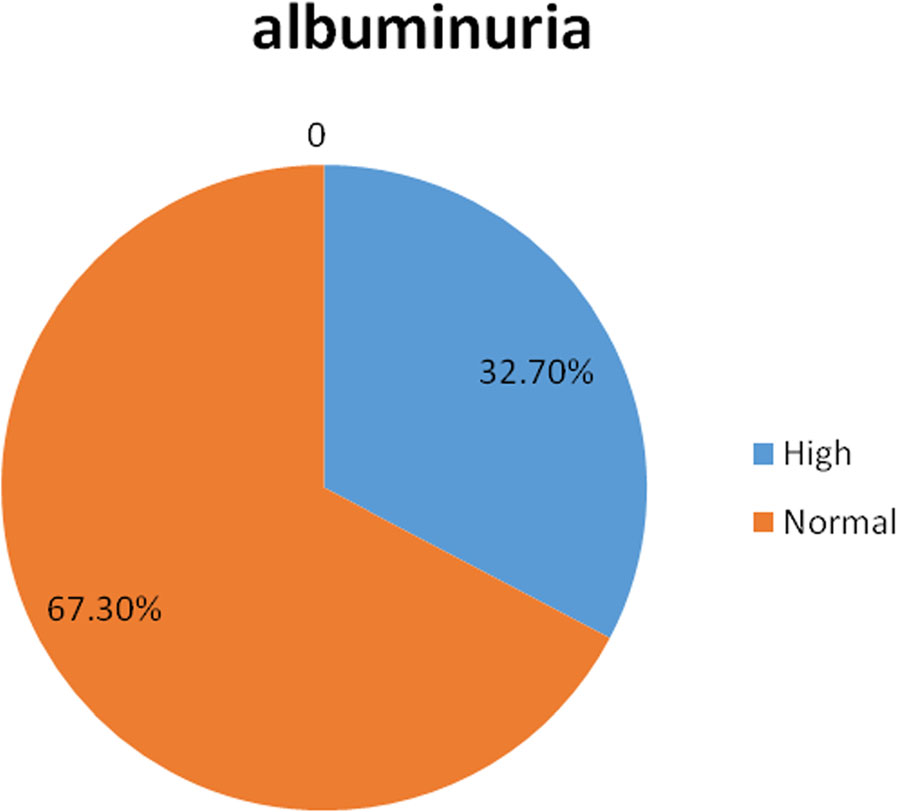
Albuminuria status of the study subjects. Albuminuria was recorded in 32.7% of the patients with no significant sex differences; 31.7% in females and 34.6% in males (*p* = 0.572)

Figure 2 depicts a pie-chart representing the prevalence of albuminuria amongst the study patients.

**Fig. 3:**
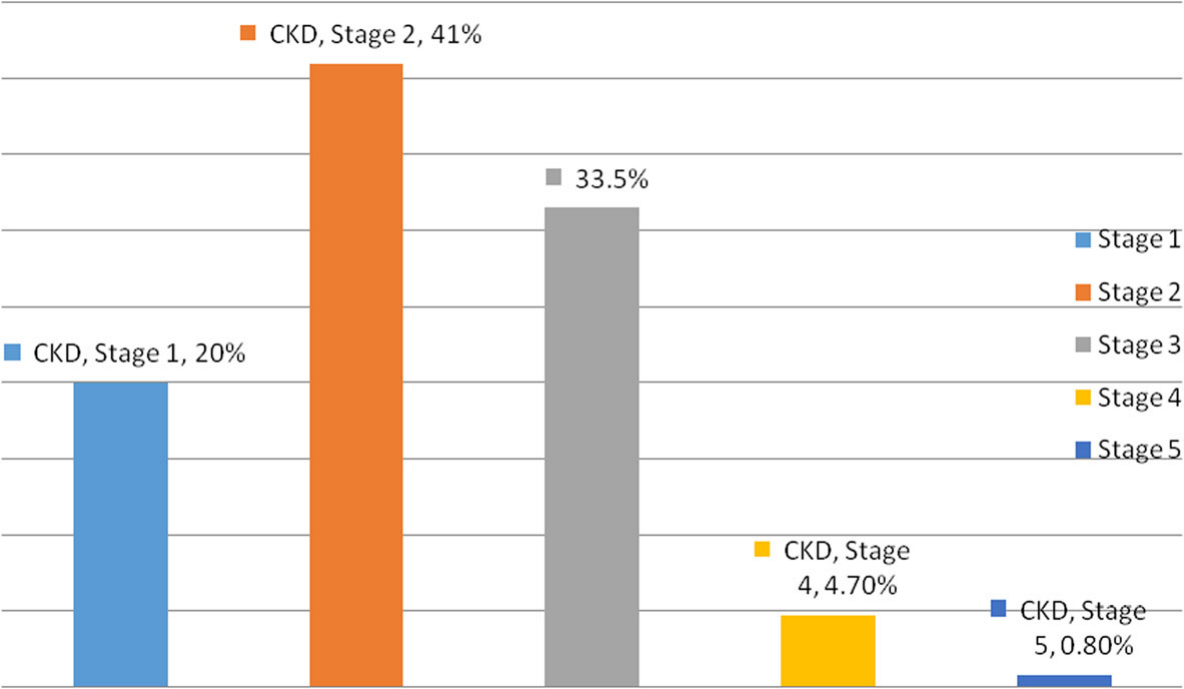
Chronic Kidney Disease/KDIGO stages and the proportions of study subjects in each stage.  There earlier stages 1 and 2 of CKD are moer prevalent than the later ones of stages 3, 4 and 5. This shows that not all stages would progress, and if some m,ay progress, the rates will vary

Figure 3 is a bar chart showing the proportion of the study patients in the various of KDIGO stages of chronic kidney disease.

## Discussion

The prevalence of CKD stages 3 to 5 in this adult population who were 30 yrs. and above, with type 2 diabetes, was 39.0% (95%CI, 34.3–44.2) by eGFR (< 60 ml/min/1.73 m^2^) and 32.7% (95%CI, 32.7–37.4) for albuminuria. Focusing on each CKD-stage, 33.5% were in stage-3, 4.7% in stage-4 and 0.8% in stage-5 in this study. The occurrence of CKD was previously unknown to both the patients and healthcare providers. These stages of CKD are often asymptomatic, so patients do not promptly seek care. The stages are only detected when appropriate tests are done.

The policy of cost-sharing in public health facilities may have limited access to both clinical care and laboratory diagnostic services of the patients who could not afford them. This study excluded any patients who had recently been discharged from hospital; therefore, the prevalence reported is an underestimate.

The meta-analysis of studies on diabetes kidney disease in sub-Saharan Africa by Noubiap JJ et al. [16] gave a summary on methods used and characteristics of the patients involved in those studies, mentioning some shortcomings that limited adequate comparability. Several of those studies included patients with type 1 and 2 diabetes, conditions with fairly divergent time paths towards evolution of kidney disease, even when risk factors may be common.

In this study, we discuss the prevalence CKD stages 3 to 5 in context of its magnitude, clinical significance, opportunities of case-finding and implication to care needs.

Studies to determine the prevalence of CKD stages 3 to 5 and/or albuminuria in patients with type 2 diabetes in sub-Saharan Africa have found a wide range of figures. Our own study on patients with type 2 diabetes for less than 2 years but relatively younger, in a tertiary hospital, found 26% had albuminuria [10]. Ngassa et al., in S. Africa found 33.6% of their patients had proteinuria and 17.3% had CKD of stages 3, 4 and 5 (eGFR**<** 60 ml/minute/1.73m^2^), aggregated for both type 1 and 2 diabetes [17]. The prevalence of CKD in patients with type 2 diabetes was 41.1% in a Nigerian study [18]. Advanced CKD, of eGFR< 60 ml/min/1.73 m^2^, was found in 18.2 and 23.8% of subjects with diabetes using the MDRD and Cockcroft-Gault (C-G) equations respectively, in an Ethiopian study [19], demonstrating that the equation used to calculate eGFR may also explain variations in prevalence. Two studies on patients with type 1 and type 2 diabetes were conducted in two different places in Tanzania: Janmohamed et al., found 79.9% had albuminuria and 83.7% had CKD (eGFR< 60 ml/minute/1.73m^2^) [20], while in Lutale et al., 17% had albuminuria and 22% had CKD stages 3 to 5 [21]. Their methods of determining proteinuria were different and target populations varied in ages, duration of diabetes and other characteristics. The prevalence of CKD in studies in sub-Saharan Africa is relatively high, in spite of the differences in patient characteristics within diabetes and the methods used to determine the eGFR. However, CKD and albuminuria still exhibit common determinants of aging, hypertension, poor glycaemic control and socio-economic challenges in studies that evaluated them. This study did not show glycaemic control as a significant predictor of chronic kidney disease, likely because of its cross-sectional design.

Screening and case finding for CKD in patients with diabetes is important because early stage disease can be managed to delay progression [22, 23].

The potential cost of morbidity and mortality from progressive CKD in type 2 diabetes is enormous, barely affordable in resource-scarce settings. This is much more important in high-risk individuals where the factors that initiate CKD, are often also the drivers of CKD progression.

Renal functions do improve when therapeutic targets are attained [24], therefore CKD and its drivers of progressions should be actively sought for in patients attending general diabetes clinics as standard of care. Modelling of data on screening strategies has demonstrated the cost-effectiveness of screening for CKD and optimally managing hypertension [25].

.Our study showed that age above 50 years, (and age as a continuous variable), was associated with having CKD/KDIGO stages 3 to 5. In addition, living with diabetes for 5 years and more, increased the odds of having advanced CKD stages. Similarly, other studies have demonstrated that aging is associated with deteriorating kidney function [26, 27], and long duration of diabetes compounds it [28]. These parameters are easily determined in clinics and should assist in stratifying our patients into risk categories during routine follow-up for case-finding, especially in patients who also have other risk factors like hypertension, chronic poor glycaemic control and cigarette-smoking. Therefore, sophisticated and/or costly tests are not necessary for case-finding of chronic kidney disease in routine care.

Hypertension, a modifiable risk factor increased by 3-fold, the odds of having advanced CKD stages in this study. Hypertension causes chronic kidney disease in type 2 diabetes [29–33] and other cardiovascular events like heart failure [34] and stroke [35, 36] but when hypertension is under control in such subjects who retain normal kidney function, it mitigates the potential for developing CKD [37, 38]. Conversely, poor or insufficient control of hypertension initiates CKD (and causes progression of CKD to more advanced stages). Use of ACEi’s/ARB’s in control of hypertension mitigates renal disease progression [39–42]**.** Our study registered 69.0% users of ACEi/ARB agents; however, we did not determine duration of use, doses nor their adherence patterns. Just below a quarter of our study patients with hypertension, at the time of enrolment, had optimal control of the high blood pressure. We have previously documented similar modest proportion of patients with hypertension on type 2 diabetes who achieved optimal Blood Pressure control while on treatment [43]. Another study elsewhere in sub-Saharan Africa [44] also reported similar small proportions, emphasizing that there is urgent need to increase the proportions achieving blood pressure targets.

From this study we infer that diabetes care in the clinic was neither sufficient to achieve optimal control of the modifiable risk factors nor able to detect complications in the patients. These findings suggest that patients with type 2 diabetes for 5-years and more, aged 50 years and above, have hypertension, also are cigarette-smokers and of modest socio-economic capacity should be screened for CKD. Many patients with type 2 diabetes who attend public hospital clinics in sub-Saharan Africa, may look well, but they will need screening for CKD. The WHO document of Wilson and Jungner [45] supported CKD screening amongst patients with type 2 diabetes because it is a public health problem, that early disease is recognizable and screening tests are acceptable to populations, amongst its 10 items. Indeed, screening for chronic kidney disease in diabetes has been recommended but in the developed world [46–48]. A review from experts to justify the same for overall CKD screen in sub-Saharan Africa was recently published. They noted that screening for a disease would demand availability of (or access to) treatment and that is our major undoing in sub-Saharan Africa [49, 50]. However, improvement in public health systems should equalize opportunities for access to quality care, in this context, of high-risk diabetic patients like these in our study. The audit part of this study has been published [11]**.** Spot urine test for albuminuria and occasional kidney function test for eGFR are not often done on these patients, as standard of care. Doing these relatively simple tests unmasked very important clinical information which had been missed out before.

## Conclusion

Screening for CKD and case-finding yielded important information on up to one-third of subjects with type 2 diabetes who were more likely to transit to ESRD or develop cardiovascular events and/or mortality. They needed to be linked to and retained in appropriate care where they are risk-stratified to enhance their level of risk factor control and monitoring.

Therefore, public health systems or hospitals should strive to improve the quality of diabetes care they offer by providing the necessary clinical and laboratory capacities that identify high-risk patients and enabling their care needs.

## Data Availability

Data and materials are available and can be found in additional file 1.
